# A single-dose chimeric Newcastle disease virus (NDV)/chikungunya virus (CHIKV) is an effective CHIKV vaccine candidate

**DOI:** 10.1128/jvi.00568-26

**Published:** 2026-07-10

**Authors:** Qiu-Yan Zhang, Yang Zhang, Zhe-Rui Zhang, Yue Li, Qi Yan, Ya-Nan Zhang, Bo Zhang

**Affiliations:** 1 State Key Laboratory of Virology and Biosafety, Wuhan Institute of Virology, Chinese Academy of Sciences 74614, Wuhan, China; 2 Department of Good Clinical Practice, Medical Guarantee Center, Second Affiliated Hospital of Naval Medical University, Shanghai, China; 3 Department of Life Sciences and Medicine, University of Science and Technology of China, Hefei, China; St Jude Children's Research Hospital, Memphis, Tennessee, USA

**Keywords:** chikungunya virus, Newcastle disease virus, vector vaccine

## Abstract

With the broad spread of the chikungunya virus (CHIKV), there is an increasing demand for more effective and protective vaccines. In this study, we first developed a Newcastle disease virus (NDV) vector vaccine candidate expressing the complete envelope (E3–E1) protein of CHIKV. The immunoelectron microscopy confirmed the presence of E2 protein in the rNDV-CHIKV virion, and the virological results showed that the expression of E1 and E2 protein was stable after 10 serial passages in embryonated chicken eggs. Moreover, we assessed its safety and immunogenicity following intramuscular (i.m.) or subcutaneous (s.c.) administration in C57BL/6 mice. The immunogenicity analyses demonstrated that a single-dose immunization elicits high titers of CHIKV E2-specific IgG antibodies, potent neutralizing activity, and T-cell immune responses. Importantly, both routes of immunization conferred complete protection against wild-type CHIKV challenge in C57BL/6 mice. These findings present a promising novel vector-based vaccine candidate with significant potential for effective CHIKV prevention.

**IMPORTANCE:**

Chikungunya virus (CHIKV) imposes a substantial global health burden, characterized by arthralgia that can persist for years, neurological complications, and potentially fatal outcomes in vulnerable populations. Currently, two vaccines against CHIKV have been approved: a live-attenuated vaccine (Ixchiq) and a virus-like particle (VLP) vaccine (Vimkunya). However, Ixchiq has been withdrawn from clinical use due to significant side effects, highlighting the urgent need for the development of novel vaccine strategies. Our research has successfully developed a novel vaccine candidate that utilizes Newcastle disease virus as a vector to express CHIKV antigens. This innovative strategy offers multiple advantages: cost-effectiveness, genetic stability of the inserted gene, and robust immunogenicity with effective immune protection. This approach offers a contribution in addressing the emerging threat posed by CHIKV.

## INTRODUCTION

Chikungunya virus (CHIKV) is an important, re-emerging mosquito-borne virus within the *Alphavirus* genus of the *Togaviridae* family. CHIKV infection typically causes a self-limiting disease known as chikungunya fever (CHIKF), which is characterized by abrupt fever, rash, and polyarthralgia (1). In the newborns, elderly, and individuals with pre-existing medical conditions, CHIKF may progress to severe illness, which can increase the risk of death (2). Since its re-emergence in 2004 in Kenya (3), CHIKV has spread to more than 100 countries in Africa, Asia, Europe, and the Americas, caused several large-scale outbreaks worldwide annually, and has become a global health threat (4). In 2018, CHIKV was included in the World Health Organization’s list of priority pathogens for vaccine research and development (5). Recently, over 9,000 cases of CHIKV infection have been reported in Guangdong Province, China, drawing significant public and official attention (6).

The CHIKV genome is a single-stranded, positive-sense RNA that encodes four nonstructural proteins (nsP1–nsP4) and five structural proteins (capsid, E3, E2, 6K/TF, and E1). Nonstructural proteins mediate RNA transcription and replication, while structural proteins constitute the virus particle. The two transmembrane glycoproteins E2 and E1 form heterodimers and assemble into spikes on the surface. Both E2 and E1 are essential for virus infection; the E2 interacts with host receptors and facilitates viral binding and attachment, and E1 mediates the fusion of the virus and endosome membranes after viral entry. It has been demonstrated that the E2 protein is the primary target of neutralizing antibodies against alphaviruses (7). Therefore, the E2 glycoprotein or full-length structural proteins are the preferred antigens for CHIKV vaccine design.

Vaccines are the most effective means to prevent viral infection and transmission. Currently, only two vaccines—a live-attenuated vaccine (Ixchiq) and a virus-like particle (VLP) vaccine (Vimkunya)—have been approved by the FDA for use against CHIKV (8, 9). However, the Ixchiq vaccine carries notable safety risks. Clinical trials have demonstrated that 10%–30% of vaccine recipients develop mild-to-moderate adverse reactions, including headache, fatigue, and myalgia. Moreover, serious adverse events (SAEs) have been observed in elderly individuals, a phenomenon potentially linked to elevated type I IFN-neutralizing autoantibodies (10, 11). In addition, measles-vectored and adenovirus-vectored vaccines are under Phase 2 or Phase 1 clinical trial evaluation, respectively (12, 13). Both measles virus and adenovirus vectors are widely used in vaccine development due to the advantages of high-efficiency expression and induction of robust immune responses. However, the existence of pre-existing immune responses in the host may also reduce the antigenicity. Thus, there is an urgent need for a new vector vaccine with high efficacy, safety, and production capacity to address the threat of CHIKV.

Newcastle disease virus (NDV), an avian paramyxovirus, is not pathogenic in humans, which avoids the issue of pre-existing immunity in the population (14). In addition, NDV can rely on the well-established production process in chicken embryo eggs without complicated cell culture, which are cost-efficient and thermostable. These characteristics make NDV a promising vaccine vector for humans. NDV has been broadly used as the vaccine vector against a variety of pathogenic viruses including SARS-CoV (15), SARS-CoV-2 (16, 17), MERS-CoV (18), Ebola virus (19), Nipah virus (20), influenza virus (21), and *orthoflavivirus* (22, 23), indicating the strong ability to accommodate foreign protein, while whether NDV vectors can be used for the development of vaccines against alphaviruses remains unknown.

Herein, using the NDV LaSota vaccine strain as the vector, we successfully constructed the recombinant rNDV-CHIKV stably expressing the envelope E3–E1 protein of CHIKV, and the immunoelectron microscopy confirmed the presence of E2 protein in the rNDV-CHIKV virus particle. Moreover, a single-dose immunization with rNDV-CHIKV through the intramuscular (i.m.) or subcutaneous (s.c.) route was sufficient to elicit robust humoral and cellular immune responses, thereby conferring complete protection to immunized mice against wild-type CHIKV challenge. In summary, our findings support the potential of the NDV vector in the development of a novel vaccine candidate against the infection of CHIKV.

## RESULTS

### Development and characterization of recombinant rNDV-CHIKV

**Fig 1:**
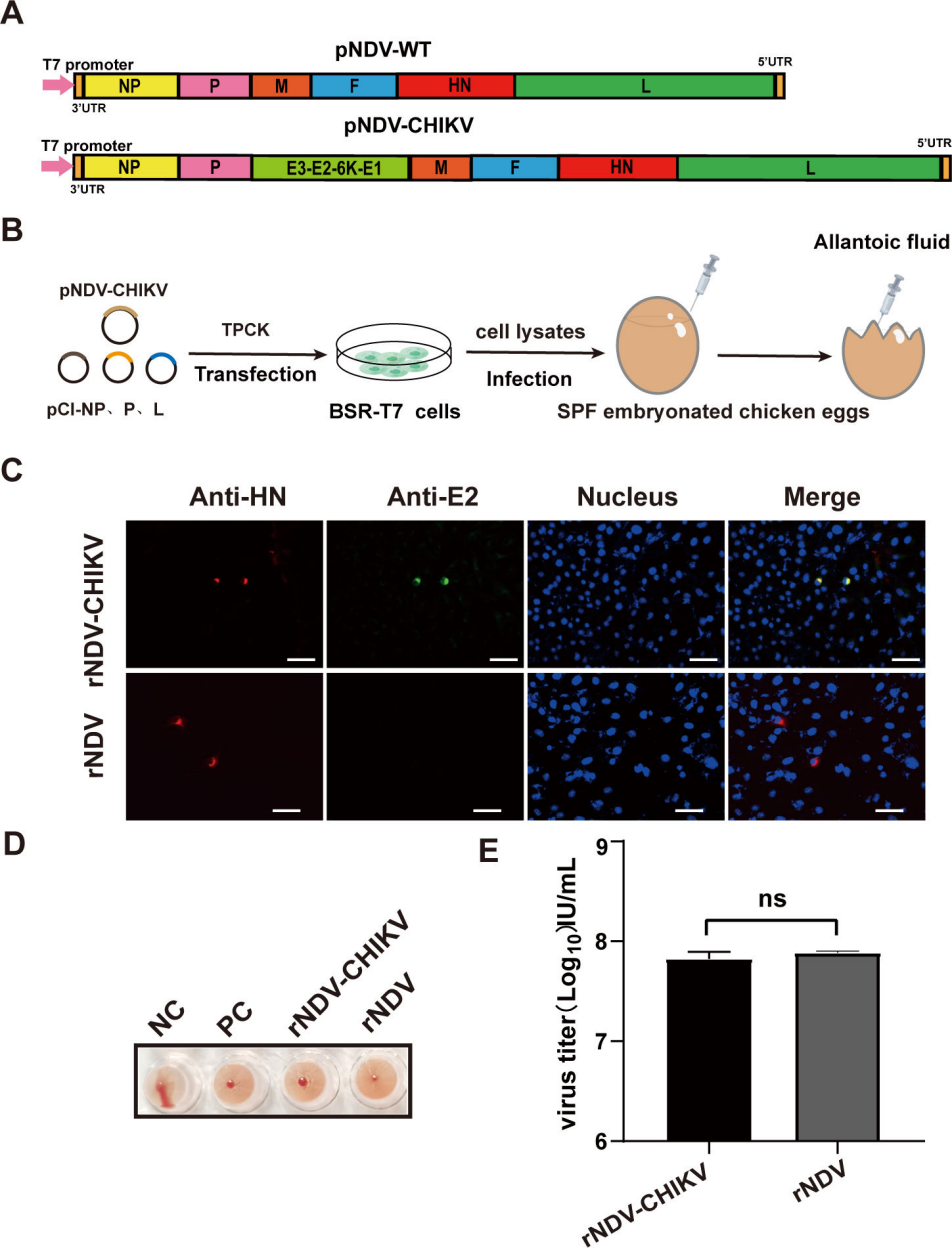
Development of recombinant rNDV-CHIKV. (**A**) The construction strategy of pNDV-CHIKV. The sequence of the full-length envelope (E3-E2-6K-E1) gene of CHIKV was inserted between the P and M genes, and noncoding regions of both 5′ and 3′ ends retained the sequences of M and P, respectively. (**B**) Schematic diagram of recombinant virus production. pNDV or pNDV-CHIKV plasmid with helper plasmids pCI-NP/P/L were transfected into BSR-T7 cells. After 48 hours post-transfection (hpt), transfected cells were lysed through repeated frozen-thaw, and cell lysis was injected into 9-day-old embryonated chicken eggs. The eggs were incubated at 37°C for 3 days, and the allantoic fluid was collected and clarified. (**C**) The expression of NDV HN or CHIKV E2 protein was detected by indirect immunofluorescence assay (IFA) in transfected cells. (**D**) The collected allantoic fluid was subjected to hemagglutination (HA) assay using 1% chicken red blood cells, and the focus formation was determined by rNDV and rNDV-CHIKV viruses. Allantoic fluid was two-fold serially diluted in PBS, and the results from the 2^6^-fold dilution are shown. NC, negative control, PBS; PC, positive control, the NDV of known titer. (**E**) Virus titers of rNDV and rNDV-CHIKV produced in embryonated chicken eggs. The rescued viruses from allantoic fluids were used to infect BHK-21 cells. The titers of each virus were determined by IFA to detect the number of HN protein-positive cells. The error bars represent standard deviation (SD).

In this study, the NDV LaSota vaccine strain was employed as the vector, and the full-length envelope protein gene (E3-E2-6K-E1) of CHIKV ECSA strain (GenBank no. MF740874.1) was inserted between the P and M genes of the antigenomic cDNA of NDV, named as pNDV-CHIKV (Fig. 1A). The inserted E3–E1 cassette forms an independent open reading frame flanked by the M-gene start (GS) and P-gene end (GE) noncoding sequences. The recombinant NDV chimera viruses expressing CHIKV E3–E1 protein were generated by co-transfection of the recombinant cDNAs with NDV helper plasmids into BSR-T7 cells and then propagated in embryonated chicken eggs (Fig. 1B). In transfected BSR-T7 cells, we observed that both HN and E2 proteins were expressed in cells from the pNDV-CHIKV group, while only the HN protein was detected in the pNDV group cells (Fig. 1C). This expression pattern confirmed the successful rescue of the rNDV-CHIKV virus. Subsequently, the cell lysate was inoculated into the allantoic cavity of chicken eggs, and the hemagglutination titer of the collected allantoic fluid was 64 hemagglutination units (HAU), comparable to that of NDV, further validating the successful production of the recombinant virus (Fig. 1D). In addition, the virus titers of rNDV-CHIKV in harvested allantoic fluid reached approximately 10^8^ FFU/mL, comparable to those of rNDV (Fig. 1E), demonstrating efficient replication of rNDV-CHIKV in embryonated chicken eggs.

**Fig 2:**
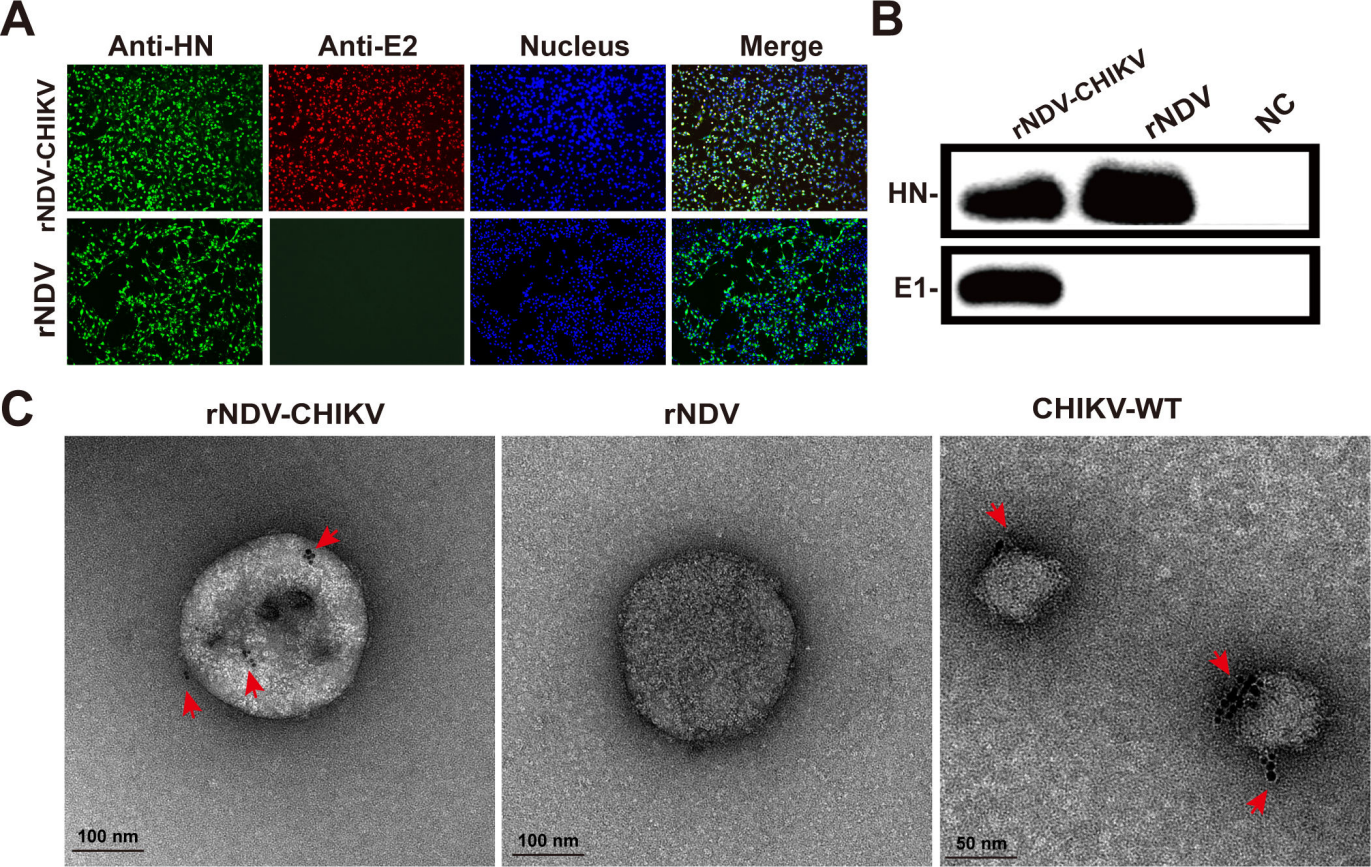
The protein expression and characterization of recombinant virus rNDV-CHIKV. (**A**) Expression of the CHIKV protein in the infected cells. BHK-21 cells were incubated with rNDV and rNDV-CHIKV for 24 h. Cells were then fixed with cold acetone, and the protein expressions were measured by IFA with anti-E2 or anti-HN antibody and observed under a fluorescence microscope. (**B**) Incorporation of CHIKV E1 into viral particles. Concentrated rNDV or rNDV-CHIKV through sucrose gradient was resolved on 12% SDS-PAGE, and the E1 and HN proteins were detected by Western blot assay. (**C**) Immunoelectron microscopy of purified rNDV and rNDV-CHIKV virus particles. Purified virus preparations were adsorbed to grids, and antigens were detected with CHIKV-specific E2 monoclonal antibodies, followed by 6-nm gold-bead-labeled secondary antibodies. The grids were subjected to negative staining and analyzed by transmission electron microscopy. The virus particle size and morphology correspond to that previously described for NDV: generally rounded particles 100 to 500 nm in diameter (21).

To confirm the expression of CHIKV protein in the recombinant viruses, BHK-21 cells were infected with rNDV-CHIKV or rNDV, and indirect immunofluorescence assay (IFA) was conducted using E2-specific antibody at 24 h post-infection (hpi). The results showed robust expression of E2 protein in rNDV-CHIKV-infected cells, but not in rNDV-infected cells (Fig. 2A). To examine whether the E1 and E2 proteins were incorporated into the NDV virions, we obtained purified viruses through sucrose density gradient centrifugation. The purified viruses were examined for the abundance of E1 and HN proteins by Western blot assay (Fig. 2B). In addition, immunoelectron microscopy further confirmed the presence of E2 protein in the rNDV-CHIKV virion, not in the rNDV virion (Fig. 2C), which may further enhance the antigenicity of the recombinant virus. In summary, these results indicated the incorporation of CHIKV E1 and E2 proteins into the recombinant virions.

### Genetic stability of rNDV-CHIKV

**Fig 3:**
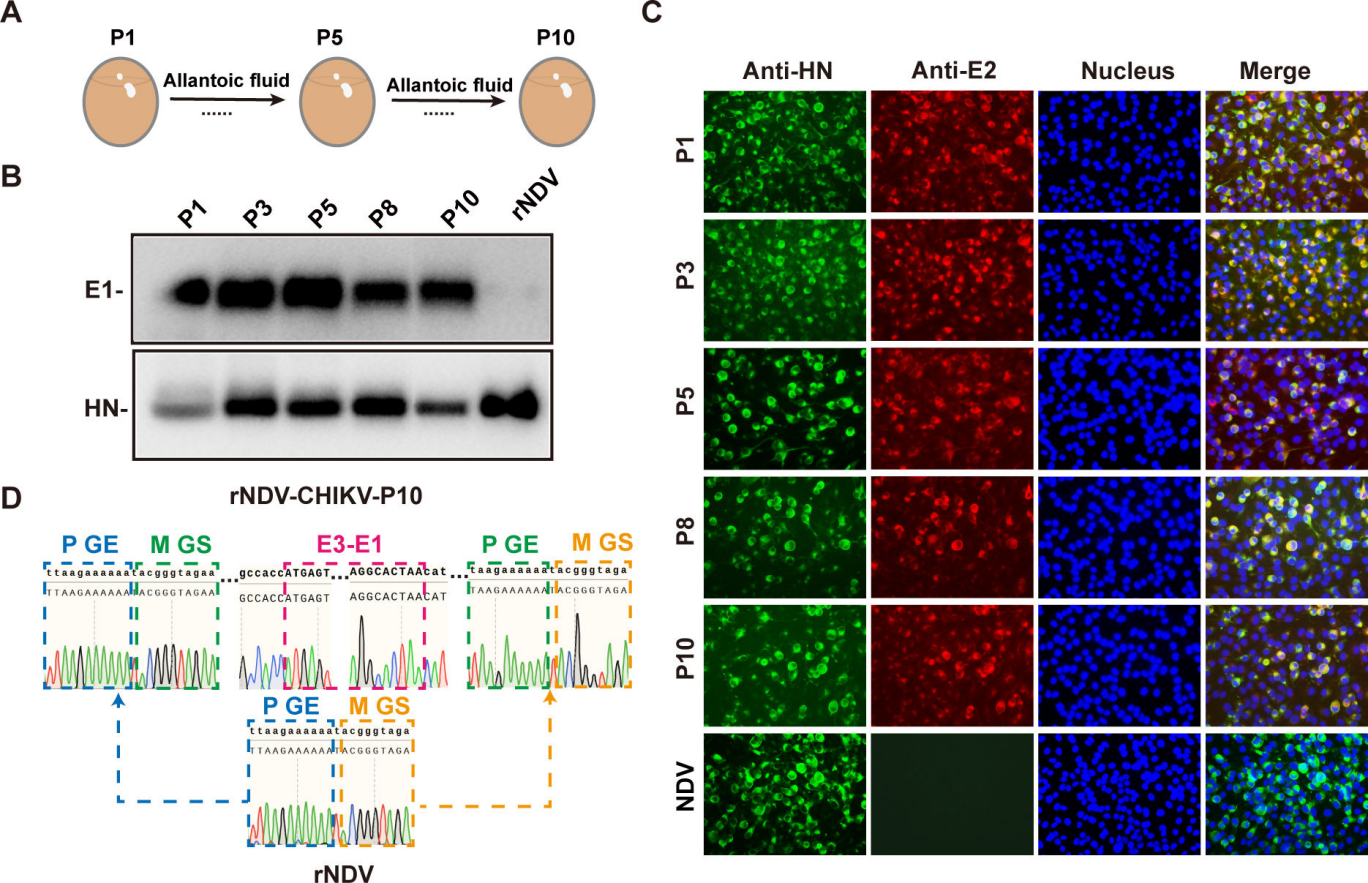
Stability assessment of rNDV-CHIKV in SPF embryonated chicken eggs. (**A**) Schematic representation of the serial passage protocol for rNDV-CHIKV in SPF embryonated chicken eggs. Allantoic fluid harvested from infected eggs was serially passaged to naive 9-day-old embryonated chicken eggs. (**B**) Western blot analysis of protein expression in passaged rNDV-CHIKV viruses. Allantoic fluid samples from passages 1, 3, 5, 8, and 10 (P1/P3/P5/P8/P10) were subjected to SDS-PAGE and immunoblotted with anti-E1 and anti-HN antibodies, respectively. (**C**) Immunofluorescence analysis of protein expression and colocalization in passaged rNDV-CHIKV. BHK-21 cells were infected with rNDV-CHIKV from passages 1, 3, 5, 8, and 10. At 24 h post-infection (hpi), cells were fixed and processed for IFA to detect the expression and localization of HN and E2 proteins. (**D**) Sequence chromatograms of RT-PCR products containing the CHIKV E3-E2-6K-E1 fragment amplified from P10 rNDV-CHIKV virus. The E3-E2-6K-E1 gene was flanked by NDV M gene start (GS) and P gene end (GE) sequences.

To validate the genetic stability of recombinant viruses, rNDV-CHIKV was serially passaged in embryonated chicken eggs (Fig. 3A). The allantoic fluid from different passages (P1/P3/P5/P8/P10) was subjected to Western blot assay, and we found that the E1 protein was stably expressed after passage (Fig. 3B). Moreover, the BHK-21 cells infected with the passaged rNDV-CHIKV also generated robust colocalized expression of E2 and HN proteins (Fig. 3C). Concurrently, sequencing results provided corroborative evidence that the passaged rNDV-CHIKV strains retained the inserted complete E3–E1 gene (Fig. 3D). These results showed that rNDV-CHIKV can stably express E1 and E2 proteins, further demonstrating their potential to be developed as vaccines.

### CHIKV-specific immune responses induced by rNDV-CHIKV in C57/BL6 mice

**Fig 4:**
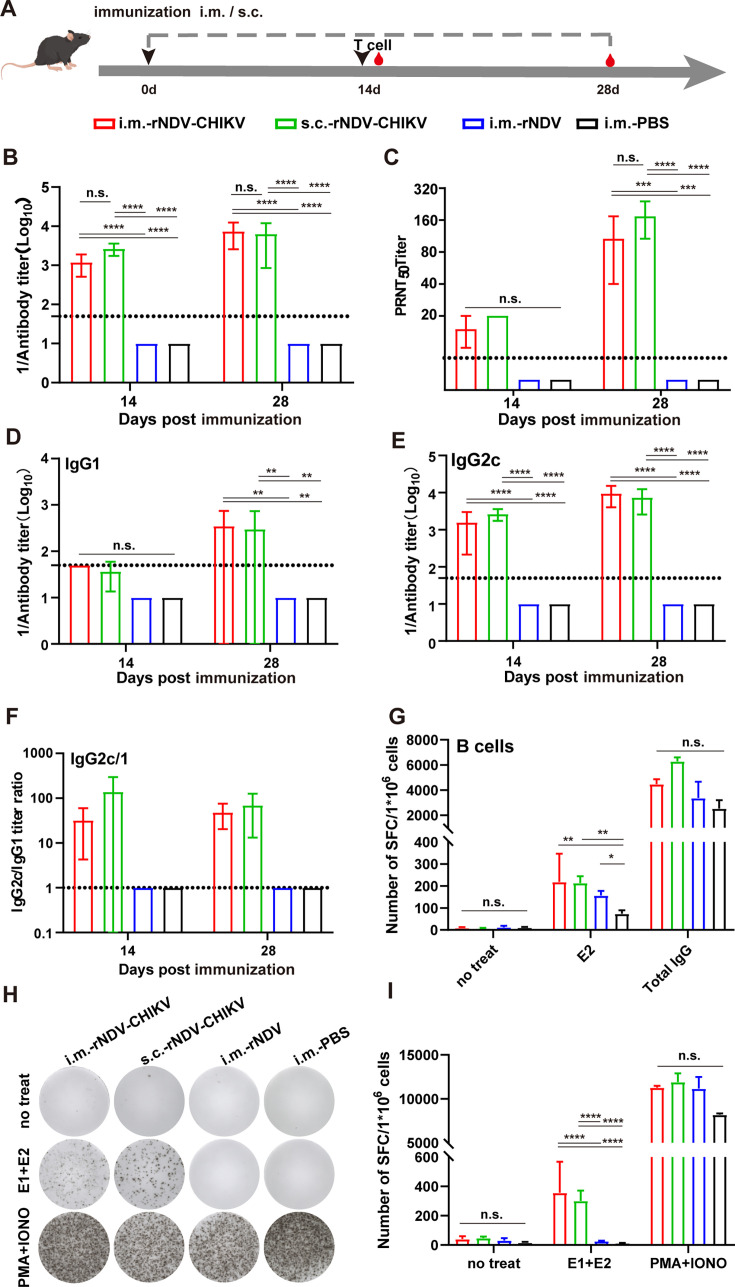
A single-dose immunization of rNDV-CHIKV induces robust humoral and cellular immune responses. (**A**) Experimental schedule of rNDV-CHIKV vaccination in C57BL/6 mice. Four- to six-week-old female C57BL/6 mice were immunized with 2 × 10^7^ IU rNDV-CHIKV via the intramuscular (i.m.) or subcutaneous (s.c.) routes, respectively. Intramuscular immunization of equivalent rNDV or PBS was set as negative controls. (**B and C**) The titers of CHIKV E2-specific antibodies (**B**) and neutralizing antibodies (**C**) from the serum samples determined by ELISA and PRNT, respectively. (**D–F**) CHIKV-E2-specific IgG subtype analysis by ELISA. The serum samples from the rNDV-CHIKV, rNDV, or PBS immunized mice were subjected to ELISA assay to measure the levels of IgG1 (**D**) and IgG2c (**E**), and IgG2c/IgG1 ratios (**F**) were calculated and presented. The dashed lines in panels **B–E** represent the limits of detection. Data were represented as the mean ± standard deviation (*n* = 3 in each group). (**G–I**) Activated B cells and T-cell responses in vaccinated mice were measured through ELISpot assay. Four groups of C57BL/6 mice (*n* = 4/group) were i.m. or s.c. inoculated with 2 × 10^7^ IU of rNDV-CHIKV, rNDV, or an equal volume of PBS. ELISpot assays detecting CHIKV E2-specific B cells and IFN-γ-secreting splenocytes were performed on day 9 post-immunization. (**G**) CHIKV-specific activated B cells were detected on E2-coated PVDF plates; anti-IgG-coated plates served as positive controls for total activated B cells. Isolated splenocytes from immunized mice were cultured for 24 h at 37°C, followed by biotinylated anti-IgG, streptavidin-ALP, and BCIP/NBT staining before spot-forming cell (SFC) enumeration. For IFN-γ detection, PMA+IONO treatment was used as a positive control, and E1+E2 represented two peptides containing 1 μg/mL E1 (HSMTNAVTI) and E2 (IILYYYELY). Representative images are shown (**H**), and the numbers of IFN-γ spot-forming cells (SFCs) were counted (**I**). The values represent mean ± SD in each group; each symbol represents a mouse. Significant differences between groups were determined using a one-way analysis of variance (ANOVA). *P < 0.05, **P < 0.01, ***P < 0.001, and ****P < 0.0001, n.s., no significant difference.

To evaluate the safety and immunogenicity of recombinant viruses, groups of C57BL/6 mice were immunized with purified rNDV-CHIKV, rNDV, or PBS through intramuscular (i.m.) or subcutaneous (s.c.) routes (Fig. 4A). The health condition of each mouse was monitored, and all mice survived and demonstrated no signs of disease. Abundant E2-specific IgG responses were elicited in all rNDV-CHIKV-immunized groups at 14- and 28- days post-immunization through the i.m. or s.c. routes. IgG titers at day 28 (i.m., 1:7,467; s.c., 1:6,400) were substantially higher than day-14 values (i.m., 1:1,200; s.c., 1:2,667), with no statistically significant discrepancy in the titers between the i.m. and s.c. immunized groups (Fig. 4B). To further test whether the antibodies have neutralizing activity, the serum of each mouse 14 and 28 days post-immunization was subjected to a plaque reduction neutralization test (PRNT) using the wild-type CHIKV virus on BHK-21 cells. As shown in Fig. 4C, consistent with the IgG antibody results, neutralizing activity was detectable in mice vaccinated with rNDV-CHIKV but absent in those inoculated with rNDV or PBS. Additionally, the neutralizing antibody titer increased from 1:20 at 14 days post-immunization to 1:106 (i.m.) or 1:173 (s.c.) at 28 days post-vaccination. Furthermore, to evaluate the quality of the induced antibodies, we had analyzed the IgG subtypes IgG2c and IgG1, which represent Th1- and Th2-type responses, respectively. We found that both IgG1 and IgG2c were significantly elicited after 14 or 28 days of immunization, but the titer of IgG2c (1:12,800) was higher than that of IgG1 (1:3,200), resulting in an IgG2c/IgG1 ratio over 1 (Fig. 4D through F), indicating a Th1-biased immune response. Moreover, the activated E2-specific B cells were detected by enzyme-linked immunospot (ELISpot) assay. The splenocytes were isolated from the spleen of immunized mice and plated in the PVDF plates coated with E2 protein or anti-IgG antibody, which was used as a positive control. As shown in Fig. 4G, the number of E2-specific spot-forming cells (SFCs) in the rNDV-CHIKV-immunized group was approximately threefold higher than that in the PBS group, while the rNDV-immunized group exhibited a twofold increase compared to the PBS group. These findings suggest that immunization with rNDV-CHIKV specifically activated E2-targeting B cells.

To further assess T-cell responses in immunized mice, ELISpot assay was performed to detect IFN-γ or IL-4 production on day 9 after immunization with rNDV-CHIKV. The splenocytes were stimulated with the two peptides corresponding to CHIKV E1 and E2 proteins. Nonspecific stimulation of PMA and ionomycin (PMA+IONO) was used as a positive control, and the medium alone served as a negative control. Much higher numbers of IFN-γ-secreting cells were observed in the rNDV-CHIKV-immunized mice compared to rNDV-immunized mice, and the SFC number of the i.m. group was slightly larger than that of the s.c. group, with no significant difference (Fig. 4H and I). Meanwhile, no obvious IL-4-secreting cells were elicited (data not shown). Taken together, our data suggested that rNDV-CHIKV could induce strong IFN-γ T-cell responses in mice after a single-dose inoculation, further confirming a Th1-biased immune response, comparable to the results of the IgG antibody subtype.

### rNDV-CHIKV produces complete protection against WT CHIKV challenge

**Fig 5:**
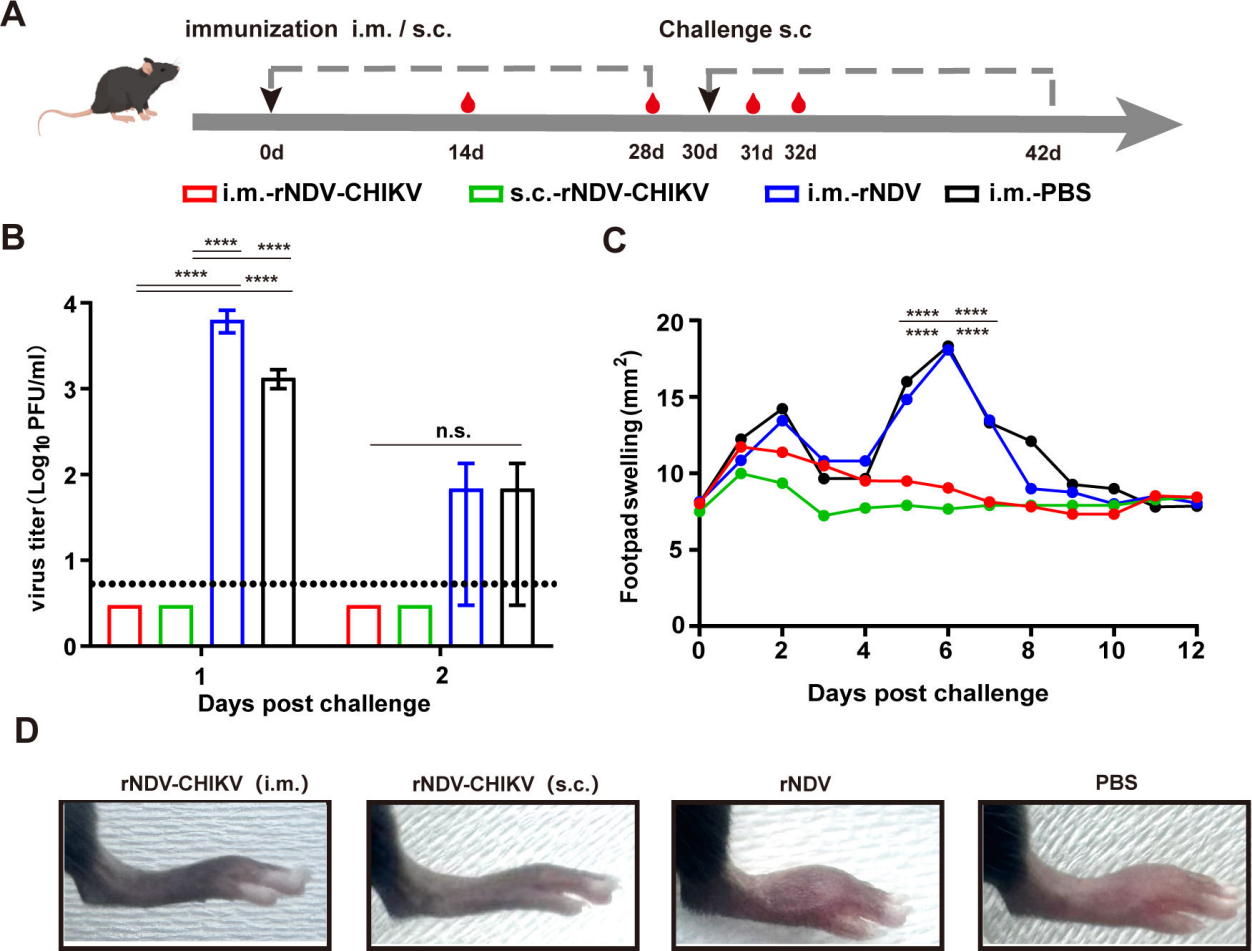
A single rNDV-CHIKV immunization completely protects C57BL/6 from wild-type CHIKV challenge. (**A**) Experimental schedule of rNDV-CHIKV vaccination and WT virus challenge in C57BL/6 mice. On day 30 post-immunization, immunized mice were challenged with 10^5^ PFU WT CHIKV. (**B**) The viremia of mice receiving a single-dose injection of rNDV-CHIKV, rNDV, or PBS, respectively, 2 days post-challenge. (**C**) The footpad swelling measurements for 12 days post-challenge. There were significant differences between the rNDV-CHIKV-immunized group and rNDV- or PBS-immunized groups. (**D**) Representative images of the injected feet of the mice from each group on day 6 post-challenge. Data represent the mean ± standard deviation of three mice at each time point in each group. Significant differences between groups were determined using a one-way analysis of variance (ANOVA). The asterisks denote statistical differences between the indicated groups. ****P < 0.0001; n.s., no statistical difference.

To evaluate the *in vivo* protective efficacy of the rNDV-CHIKV against CHIKV infection, the C57BL/6 mice immunized with rNDV-CHIKV, rNDV, or PBS were challenged with 10^5^ PFU of WT CHIKV via subcutaneous (s.c.) injection on day 30 post-vaccination. Viremia was monitored for a 2-day period, while footpad swelling was assessed over a 12-day observation period following viral challenge (Fig. 5A). As shown in Fig. 5B, viremia was induced in mice from both the PBS and rNDV-immunized groups, persisting through 2 days post-infection, whereas no virus was detected in rNDV-CHIKV-immunized mice. Additionally, PBS-immunized mice exhibited significant footpad swelling, with two distinct peaks occurring at approximately 2 and 6 days post-challenge, respectively, with full recovery by day 12 post-challenge (Fig. 5C and D). In contrast, rNDV-CHIKV-immunized mice developed only minimal footpad swelling shortly after initial infection, which subsided rapidly—demonstrating strong protective efficacy. Collectively, these findings demonstrated that a single rNDV-CHIKV immunization confers complete protection against CHIKV infection.

## DISCUSSION

Climate change, increased urbanization, and frequent global travel have greatly expanded the distribution range of mosquito-transmitted CHIKV and made it a major global health threat. Vaccination is the most powerful method to control virus infection. In this study, using NDV vaccine strain LaSota as the backbone, we constructed a new vector vaccine candidate, rNDV-CHIKV, which could stably express CHIKV E3–E1 protein after serial passage in embryonated chicken eggs. Moreover, a single-dose immunization of rNDV-CHIKV in C57BL/6 mice could induce strong humoral and cellular immunity and provide complete immune protection against WT CHIKV challenge. Our study provided proof of using the NDV vector as a new strategy for CHIKV vaccine design.

NDV mainly infects birds and does not infect mammals, including humans. According to their pathogenicity and virulence in chickens, NDV strains can be divided into three types: lentogenic (low virulence), mesogenic (moderate virulence), and velogenic (high virulence) (24). Avirulent NDV strains are highly safe in avian and non-avian species, and LaSota and B1 strains are usually used as vaccine vectors. In addition, NDV has been extensively researched as an oncolytic virus, with a long history of clinical trials (25). Several recombinant NDV-vectored SARS-CoV-2 vaccine candidates have undergone clinical phase I/II trials for human use, which further demonstrated the safety and efficacy (26). A clinical trial conducted in Mexico (NCT04871737) demonstrated that the NDV-vectored vaccine candidate AVX/COVID-12-HEXAPRO exhibited superior safety and tolerability profiles compared to mRNA or adenovirus-vectored vaccines. Additionally, two intramuscular (i.m.) administrations of this candidate vaccine induced robust antibody and cellular immune responses (27). Although no vaccines utilizing this viral vector have yet received approval for human use, these studies highlight the potential of this vector platform for vaccine development.

It was known that E2 protein is the primary target of neutralizing antibodies against alphaviruses. However, previous studies have demonstrated that adenovirus vectors or Modified Vaccinia virus Ankara (MVA) expressing the complete envelope protein cassette (E3-E2-6K-E1) induce substantially higher levels of neutralizing antibodies compared to vectors expressing the E2 protein alone (28, 29). Based on these findings, we inserted the E3-E2-6K-E1 polyprotein gene into the NDV genome as our target antigen. Consistent with our hypothesis, the resulting recombinant virus (rNDV-CHIKV) efficiently expressed both E1 and E2 proteins, and a single immunization dose induced robust IgG and neutralizing antibody responses. Furthermore, immunoelectron microscopy analysis confirmed the presence of E2 protein on the surface of purified viral particles (Fig. 2C), indicating that this system has the potential to further improve vaccine efficacy.

In addition, genetic stability represents a critical challenge in the development of viral vector-based vaccines. To evaluate the genetic and functional stability of rNDV-CHIKV, we performed serial passaging in embryonated chicken eggs for 10 consecutive rounds. Our findings demonstrated that rNDV-CHIKV maintained stable replication and transgene expression through at least 10 passages, with no detectable genetic instability or reduction in protein expression levels (Fig. 3). These results indicate a low risk of transgene deletion in rNDV-CHIKV, thereby supporting its potential utility as a stable and effective vaccine candidate.

For immune responses, we found that rNDV-CHIKV immunization by the i.m. or s.c. routes in C57BL/6 mice generated a Th1-skewed immune response with the IgG2c/IgG1 ratio >1 (Fig. 4). Previous studies have suggested that Th1 (Type 1 T-helper) responses, crucial for eliminating intracellular pathogens, effectively activate immune cells and complement and bind more strongly to Fc receptors than Th2-associated antibodies, enhancing pro-inflammatory antiviral immunity. Moreover, rNDV-CHIKV immunization also induced strong IFN-γ T-cell responses with antigen-specific peptide stimulation, and there were no significant differences between two immunization routes, which may be probably needed to maintain long-term immunity.

Collectively, we have successfully developed a stable recombinant NDV vector vaccine candidate (rNDV-CHIKV) that efficiently expresses the CHIKV envelope protein. This vaccine candidate induced robust humoral and cellular immune responses, conferring complete protection in C57BL/6 mice against challenge with WT CHIKV via both i.m. and s.c. routes of administration. These findings highlight the promising potential of NDV-based vector platforms for the development of novel, effective vaccines against CHIKV infection.

## MATERIALS AND METHODS

### Cell cultures, viruses, and antibodies

BSR-T7 cell lines and BHK-21 cells were cultured at 37°C with 5% CO₂_2_ in Dulbecco’s modified Eagle’s medium (DMEM; Gibco, Thermo Fisher Scientific, America) with 10% fetal bovine serum (FBS), 100 U/mL penicillin, and 100 mg/mL streptomycin. Moreover, BSR-T7 cells were maintained in medium with 1 mg/mL of G418. The CHIKV ECSA strain (GenBank no. MF740874.1) was propagated from the virus stock, which was isolated from a Pakistan patient during the virus outbreak in 2016–2017, on Vero cells (30). The polyclonal antiserum against CHIKV E2 or E1 protein was produced by immunizing the mouse with the recombinant E2 or E1 protein in our lab. Monoclonal E2 antibody was purchased from SinoBiological (China). The fluorescein isothiocyanate (FITC)-conjugated goat anti-mouse IgG antibody, the Alexa Fluor 488-anti-mouse/568-anti-rabbit IgG, and horseradish peroxidase (HRP)-conjugated goat anti-mouse IgG, IgG1, and IgG2c were purchased from Proteintech (China), and the goat anti-mouse IgG conjugated to 6-nm gold beads was purchased from Abcam (Britain).

### Plasmid construction

The full-length antigenomic cDNA of NDV LaSota vaccine strain (GenBank no. AF077761) was kindly provided by Zhejiang Difference Biotechnology, Co. Ltd. and used as the backbone to construct the pNDV-CHIKV clones. The full-length envelope gene (E3-E2-6K-E1) of the CHIKV ECSA strain (GenBank no. MF740874.1) was inserted into the cDNA of NDV LaSota between P and M genes. For the construction of the pNDV-CHIKV clone, three PCR fragments of upstream noncoding sequences of M gene start (GS), full length of E3-E2-6K-E1, and downstream noncoding sequences of P gene end (GE) were amplified and fused, followed by digestion by R*sr*II and M*lu*I restriction sites and ligation into the cDNA clone of pNDV-WT. To enhance the expression of the E3-E2-6K-E1 gene, the Kozak sequence (gccacc) was inserted before the ATG start codon. The construct was verified by DNA sequencing.

### Rescue of rNDV and rNDV-CHIKV viruses

At the day before transfection, 3 × 10^5^ BSR-T7 cells were seeded in 35-mm dishes. The next day, a transfection cocktail containing 2 µg of pNDV-WT or pNDV-CHIKV, 1 µg of pCI-NP, 0.5 µg of pCI-P, 0.5 µg of pCI-L, and 10 µL of Fugene HD (Promega) in 300 µL of Opti-MEM (Gibco) was gently mixed by pipetting and incubated at room temperature (RT) for 15 min. Then, the transfection complex was added dropwise to each dish, and the cells were incubated at 37°C with 5% CO_2_. At 48 h post-transfection (hpt), the transfected cells were collected in PBS, followed by freeze-thawing three times at −80°C. Supernatants from the freeze-thawed cells were obtained and injected into the allantoic cavity of 9-day-old specific pathogen-free (SPF) embryonated chicken eggs. The eggs were incubated at 37°C for 3 days before being cooled at 4°C overnight. The allantoic fluid was collected and clarified by low-spin centrifugation to remove the debris. The presence of the rescued rNDV and rNDV-CHIKV viruses was determined by hemagglutination (HA) assay using 1% chicken red blood cells.

### Hemagglutination (HA) assay

For the HA assay, 2-fold serial dilutions of the allantoic fluids were prepared in 96-well microtiter plates by mixing 50 μL of phosphate-buffered saline (PBS) with 50 μL of the allantoic fluids in each well. Subsequently, 50 μL of 1% chicken red blood cells was added to each well, and the plates were incubated at room temperature for 30 min before the results were recorded. The hemagglutination titer was defined as the highest dilution of the sample that induced red blood cell agglutination.

### Indirect immunofluorescence assay (IFA)

The passaged rNDV or rNDV-CHIKV allantoic fluids were fixed at 24 h post-infection (hpi) using cold acetone in methanol (5%) for 10 min and washed with PBS three times. Then, the fixed cells were incubated with anti-E2 (1:200) and anti-HN (1:200) antibodies for 1 h. After washing with PBS three times, the cells were incubated with Alexa Fluor 488-anti-mouse and 568-anti-rabbit IgG antibodies at room temperature for another 1 h. After three PBS washes, the slides were mounted with 95% glycerol to examine under a fluorescence microscope.

### Virus titration

The stocks of rNDV or rNDV-CHIKV were titered using a modified monolayer plaque experiment. Briefly, a series of 10-fold dilutions were prepared by diluting 15 μL virus stock with 135 μL DMEM containing 2% FBS, and 100 μL of each dilution was seeded into 24-well plates containing confluent BHK-21 cells. Infected cells were incubated at 37°C for 1 h, and the medium containing 2% FBS was overlaid. After incubation for 24 h, the cells were fixed and detected by IFA, as described above. The viral titer was calculated as focus-forming units (FFU)/mL.

### Virus concentration and purification

Allantoic fluids were clarified by centrifugation at 3,441 × *g* at 4°C for 30 min. Viral particles were then pelleted through a 20% sucrose cushion in NTE buffer (100 mM NaCl, 10 mM Tris–HCl, and 1 mM ethylenediaminetetraacetic acid [EDTA], at pH 7.4) via ultracentrifugation at 26,500 rpm for 2 h at 4°C using a SW32 rotor (Beckman Coulter, Brea, CA, USA). Following aspiration of supernatants, virus pellets were resuspended in phosphate-buffered saline (PBS, pH 7.4). The resuspended virus was subsequently layered onto a 20%–60% linear sucrose gradient prepared in PBS and subjected to ultracentrifugation at 28,000 rpm for 3 h at 4°C using a SW41 rotor (Beckman Coulter, Brea, CA, USA). Purified virus fractions were then carefully harvested from the gradient.

### Immunoelectron microscopy

Purified virus particles were adsorbed to 200-mesh Formvar-/carbon-coated nickel grids. For immunolabeling studies, grids were blocked in 4% bovine serum albumin (BSA) dissolved in PBS and incubated with the anti-CHIKV E2 monoclonal antibody. Grids were washed in PBS and incubated with goat anti-mouse IgG conjugated to 6-nm gold beads (Abcam, Britain). The grids received a final wash, followed by negative staining with 1% ammonium molybdate. Grids were examined under a model Talos L120C transmission electron microscope (Thermo Scientific, USA) at 120 kV.

### Western blot assay

Concentrated virus samples were heated at 95°C for 10 min and analyzed by SDS-PAGE (12 %). The proteins were then transferred onto a 0.2-μm PVDF membrane (Bio-Rad), followed by blocking with 5% skim milk in TBST buffer and sequentially incubating with anti-NDV HN (1:1,000), anti-CHIKV E1 (1:2,000) antibodies, and secondary HRP-conjugated anti-mouse/rabbit IgG antibodies (1:2,000). After three washes with TBST buffer, the signals were detected with a chemiluminescence system (ChemiDoc, Bio-Rad).

### Mouse experiments

Studies related to rNDV and rNDV-CHIKV infection were performed in an animal Biosafety Level 2 (ABSL-2) facility, and studies related to WT CHIKV infection were performed in a Biosafety Level 3 (BSL-3) facility at Wuhan Institute of Virology under a protocol approved by the Laboratory Animal Ethics Committee of Wuhan Institute of Virology, Chinese Academy of Sciences (Permit number: WIVA26202406). All mice were bred and kept under specific pathogen-free conditions and cared for following the recommendations of the National Institutes of Health Guidelines for the Care and Use of Experimental Animals. Groups of 4- to 6-week-old female C57BL/6 mice (*n*=3 or 4 per group) were immunized with 2 × 10^7^ IU concentrated rNDV-CHIKV viruses via intramuscular (i.m.) or subcutaneous (s.c.) routes, respectively. An equal amount of rNDV or PBS was immunized by the i.m. route as negative controls. The immunized mice were monitored daily for survival and health condition. At 14 or 28 days post-immunization, the titers of total anti-CHIKV IgG antibodies and neutralizing antibodies in serum were examined by ELISA and PRNT, respectively. At 30 days post-immunization, mice were challenged s.c. with 1 × 10^5^ PFU of CHIKV ECSA strain (GenBank no. MF740874.1). Viremia was quantified by plaque assay on day 1/2 post-challenge, and foot swelling was monitored for 12 days.

### Enzyme-linked immunosorbent assay (ELISA)

ELISA was used to detect the titers of CHIKV-specific IgG antibodies from the serum samples collected from the immunized mice. The 96-well microtiter plates were coated with E2 protein (SinoBiological, #40440-V08B-100, 1 μg/mL, and 100 μL/well) at 4°C overnight and then blocked with 5% skim milk at 37°C for 2 h. Sera from the immunized mice were 4-fold diluted with 2% skim milk and added to plates for 2-h incubation. After washing steps, HRP-conjugated goat anti-mouse IgG, IgG1, or IgG2c antibody (1:5,000) was added for 1 h at 37°C. The plates were then incubated with a mixed substrate (two-component 3,3’,5,5’-tetramethylbenzidine [TMB] color development kit [Proteintech]). The optical density at 450 nm was detected and analyzed by a multimode microplate reader (Varioskan Flash; Thermo Fisher) after the addition of 1 M H_2_SO_4_ to terminate the coloration reaction. The antibody titers were defined as the highest dilution of sera giving an optical density twice that of the naive serum.

### Plaque reduction neutralization test (PRNT)

Briefly, approximately 100 PFU of WT CHIKV was preincubated with 2-fold serial dilutions of heat-inactivated mouse sera (starting at 1:8 dilution) for 1 h at 37°C, and then the mixture was added to BHK-21 cell monolayers in 12-well plates and removed after 1 h of incubation, followed by plaque assay to quantify viral titers. After 2–3 days of cultivation, the cells were fixed with 3.7% formaldehyde for 24 h and stained with 1% crystal violet for 10 min. The neutralization titers were determined to be the highest dilutions for which the virus plaque count was reduced by 50% compared with control.

### Enzyme-linked immunospot (ELISpot) assay

To detect CHIKV E2-specific activated B cells or IFN-γ- or IL-4-secreting splenocytes, ELISpot assays were performed.

For activated B cell detection, PVDF microplates (Millipore) were pre-coated with 15 μg/mL recombinant CHIKV E2 protein (SinoBiological, China), whereas parallel plates coated with anti-mouse IgG antibody were used as positive controls. Splenocytes were freshly isolated from rNDV-CHIKV-immunized mice on day 9 and seeded into the pre-coated plates, followed by incubation at 37°C for 24 h. After thorough washing to remove unbound cells and impurities, the plates were incubated with biotin-conjugated anti-IgG antibody and streptavidin-conjugated alkaline phosphatase (Streptavidin-ALP) (Mab Tech, Cat#3825-2A). Subsequently, the BCIP/NBT chromogenic substrate was added to each well for color development, and the spots were formed, scanned, and quantified.

IFN-γ or IL-4 ELISpot assay was performed using a commercial mouse IFN-γ or IL-4 ELISpot kit (Dakewe, China; catalog numbers 2210006 and 2210403) according to the manufacturer’s instructions. Briefly, freshly isolated splenocytes from rNDV-CHIKV-immunized mice on day 9 were plated and stimulated *ex vivo* with mixed E2- and E1-derived peptide pools (E1: HSMTNAVTI; E2: IILYYYELY) (1 μg/mL) or PMA (500 ng/mL) plus ionomycin (10 μg/mL). The peptides with purity exceeding 95% were synthesized in Motif Biotech company, China. Following incubation for 24 h at 37°C under 5% CO₂, plates were washed and incubated with biotinylated anti-IFN-γ or anti-IL-4 antibody for 1 h at 37°C. Subsequently, plates were incubated with HRP-conjugated streptavidin for 1 h at 37°C. IFN-γ or IL-4 spots were developed using the AEC substrate as per the manufacturer’s protocol. After drying, spots were visualized, scanned, and enumerated using an ELISpot image analyzer (Bio-Sys, Karben, Germany). The average number of IFN-γ spot-forming cells (SFCs) per well from duplicate cultures was calculated by subtracting the average background response from negative control wells.

### Statistical analysis

All the data were analyzed using GraphPad Prism 8.0.2 software and expressed as mean ± standard deviation (SD). The statistical significance was assigned when *P* values were <0.05. Significant differences between multiple groups were determined using a one-way analysis of variance (ANOVA).

## Data Availability

All data supporting the findings of this study are available within the article or from the corresponding author upon reasonable request.
